# Next-Generation Sequencing Sheds Light on the Natural History of Hepatitis C Infection in Patients Who Fail Treatment

**DOI:** 10.1002/hep.27192

**Published:** 2014-07-30

**Authors:** Tamer Abdelrahman, Joseph Hughes, Janice Main, John McLauchlan, Mark Thursz, Emma Thomson

**Affiliations:** 1 MRC-University of Glasgow Centre for Virus ResearchGlasgow, UK; 2 Department of Medicine, Imperial College NHS TrustLondon, UK

## Abstract

High rates of sexually transmitted infection and reinfection with hepatitis C virus (HCV) have recently been reported in human immunodeficiency virus (HIV)-infected men who have sex with men and reinfection has also been described in monoinfected injecting drug users. The diagnosis of reinfection has traditionally been based on direct Sanger sequencing of samples pre- and posttreatment, but not on more sensitive deep sequencing techniques. We studied viral quasispecies dynamics in patients who failed standard of care therapy in a high-risk HIV-infected cohort of patients with early HCV infection to determine whether treatment failure was associated with reinfection or recrudescence of preexisting infection. Paired sequences (pre- and posttreatment) were analyzed. The HCV E2 hypervariable region-1 was amplified using nested reverse-transcription polymerase chain reaction (RT-PCR) with indexed genotype-specific primers and the same products were sequenced using both Sanger and 454 pyrosequencing approaches. Of 99 HIV-infected patients with acute HCV treated with 24-48 weeks of pegylated interferon alpha and ribavirin, 15 failed to achieve a sustained virological response (six relapsed, six had a null response, and three had a partial response). Using direct sequencing, 10/15 patients (66%) had evidence of a previously undetected strain posttreatment; in many studies, this is interpreted as reinfection. However, pyrosequencing revealed that 15/15 (100%) of patients had evidence of persisting infection; 6/15 (40%) patients had evidence of a previously undetected variant present in the posttreatment sample in addition to a variant that was detected at baseline. This could represent superinfection or a limitation of the sensitivity of pyrosequencing. *Conclusion*: In this high-risk group, the emergence of new viral strains following treatment failure is most commonly associated with emerging dominance of preexisting minority variants rather than reinfection. Superinfection may occur in this cohort but reinfection is overestimated by Sanger sequencing. (Hepatology 2015;61:88–97)

An estimated 185 million people have been infected with hepatitis C virus (HCV) around the world and more than 350,000 people die from HCV-related liver diseases every year.^1^ Shared routes of transmission mean that human immunodeficiency virus (HIV)/HCV coinfection is common, affecting at least 4-5 million individuals.^2^ Advances in treatment options for HCV are rapidly emerging; such direct-acting antiviral (DAA) therapies are genotype- and subtype-specific and therefore an understanding of the total population of HCV variants present in an infected host is likely to be of increasing importance in order to identify the most appropriate treatment for infected individuals.^3^

In the last decade, an increase in the incidence of acute HCV infection among HIV-infected men who have sex with men (MSM) in Europe, Australia, and the United States has led to a substantial number of studies on this new public health problem.^4^ Descriptions of HCV reinfection in this group have been widely reported but have not employed deep sequencing or detailed phylogenetic analysis.^5^,^6^ We have previously shown that HCV in HIV-infected MSM is commonly associated with the presence of multiple strains in individual infected patients (10% with multiple genotypes). Such variation occurs as a consequence of the transmission of multiple HCV strains either around the time of initial infection or sequentially over time. We hypothesized that reinfection rates following treatment may be overestimated by standard sequencing techniques due to a lack of detection of varying dominance of minority variant strains present at the onset of infection.^3^

We used viral load monitoring and deep sequence analysis to dissect the different causes of treatment failure using pre- and posttreatment plasma samples taken from patients who failed standard of care therapy with 24-48 weeks of pegylated interferon alpha (IFN) and ribavirin (RBV).^7^ Sustained virological response (SVR) rates are typically lower in this group (59-71%) than in HCV monoinfected patients (98%).^8^,^9^ We used detailed phylogenetic analysis of pre- and posttreatment variants obtained using Sanger sequencing, clonal analysis, and next-generation sequencing (NGS) to differentiate relapse from reinfection.

## Materials and Methods

### Patient Cohort

In all, 99 patients with HIV were treated for acute HCV infection between 2005 and 2012 in a single center (the St Mary's Acute Hepatitis C Cohort). Pegylated IFNα and RBV were administered for 24-48 weeks according to British HIV Association guidelines.^7^ A group of 15 patients failed to respond to treatment, including six null responders, three partial responders, and six relapsers. Paired samples from each patient pre- and posttreatment were analyzed. Informed consent in writing was obtained from each patient and the study protocol conformed to the ethical guidelines of the 1975 Declaration of Helsinki and ethical approval was granted by the Riverside Research Ethics Committee.

### Definitions

We used viral load monitoring and phylogenetic analysis to define distinct groups of patients.

#### Patient Groups Based on Viral Load Monitoring

*Sustained virological response*: undetectable HCV RNA 24 weeks after the end of treatment. *Null response*: a reduction of less than 2 log_10_ in HCV RNA by week 12 of treatment. *Partial response*: a reduction of 2 log_10_ or more in HCV RNA by week 12, but failure to achieve an undetectable viral load. *Relapse*: an undetectable HCV RNA level at the end of treatment but reelevation of viral load within 24 weeks after the end of treatment.

#### Patient Groups Based on Phylogenetic Analysis

Sequences obtained from paired samples pre- and posttreatment were considered similar or different based on two criteria, 1) phylogenetic signal, monophyletic or nonmonophyletic lineage; 2) genetic distance >10% between sequences; this was calculated as the pairwise distance between aligned sequences.

*Persistent infection*: the presence of at least one variant present in the pretreatment sample persisting after exposure to treatment. This could be associated with new dominance; the outgrowth of a minority strain present in the pretreatment sample, or the presence of a new variant representing superinfection or a preexisting undetected variant.

*Reinfection*: the presence of new variant(s) in the posttreatment sample with no evidence of similar preexisting strains.

### Amplification and Sequencing of the E2 HVR-1 Region

Plasma stored at −80°C was thawed on ice. RNA was extracted using a QIAamp Viral RNA kit (Qiagen) as recommended by the manufacturer. cDNA synthesis was performed using SuperScript III Reverse Transcriptase (Invitrogen). Amplification of a 220 basepair (bp) region including the E2 hypervariable region-1 (HVR-1) was carried out using nested polymerase chain reaction (PCR) using a combination of genotype-specific primers as previously described.^10^ The HVR-1 has the highest degree of heterogeneity within the HCV genome and can therefore be used to differentiate HCV strains. PCR products were run on a 2% agarose gel containing SYBR safe DNA gel stain (Life Technologies) with lane markers and a 100-bp small fragment ladder (Fermentas). DNA bands were visualized under ultraviolet light and bands of appropriate size were extracted and purified using a GeneJet extraction kit (Fermentas). PCR product was sent for direct Sanger sequencing and was also cloned into the TOPO-4 vector (Invitrogen, Paisley, UK) and sent to Beckman Coulter Genomics for miniprep and Sanger sequencing in 96-well plates.

### Sanger Sequencing Analysis

Chromatograms were checked for miscalled nucleotides by visual inspection of chromatograms using BioEdit v. 7.1.3 software. Sequences were aligned using MUSCLE^11^ and maximum likelihood phylogenetic trees constructed using MEGA 5.0.^12^ Trees were generated following gap exclusion and corrections for multiple substitutions were performed using the Kimura two-parameter substitution model.^13^ The statistical robustness and reliability of the branching order within each phylogenetic tree was confirmed by bootstrap analysis using 1,000 replicates. Bootstrap values >70% were considered reliable. Sequences generated as part of this study have been submitted to GenBank.

### Next-Generation Sequencing of Pretreatment Samples

Primer binding sites for deep sequencing as well as multiplex identifiers (MIDs) for sample bar coding were synthesized into the inner primer design to create fusion primers compatible with the 454 platform. Amplicons were diluted to create a multiplexed library with equimolar concentrations. The library was sent for 454 FLX second-generation sequencing by Beckman Coulter Genomics, USA.

Sequences were de-multiplexed using a custom Perl script that identified the forward and reverse barcodes allowing one mismatch in the reverse barcode. All scripts are available on Github (https://github.com/josephhughes/HCVtoolbox). Each read was compared to a reference set of sequences from the Los Alamos HCV database and was quality checked by comparing it in a pairwise alignment to the best reference match.^14^ A read was excluded from the dataset if it had mutations relative to the reference below a Phred score of 25, and if there was only a single copy of the read. The final set from each patient was then aligned against the complete reference set of sequences using MAFFT.^15^ In order to estimate the error rate of our sequencing approach, a plasmid containing HVR-1 was sequenced using a 454 approach following endpoint dilution.

All valid reads were clustered using CD-HIT with a parameter of similarity of 90% to assign different variants detected in each sample.^16^ These variants were aligned with posttreatment variants detected by clonal analysis and reference sequences of different genotypes from the Los Alamos HCV database using MUSCLE^11^ and maximum likelihood phylogenetic trees were constructed using MEGA 5.0 as described above.^12^ All sequences generated were submitted to the European Nucleotide Archive (ENA), study accession number PRJEB4613.

## Results

### 

#### Error Rates

An alignment of control HVR-1 plasmid pyrosequences resulted in a mean depth of 16,918 at each nucleotide site. Assuming that the most frequently found sequence was the real sequence, we calculated the error rate as 0.002 per bp. This error included any error in the sequencing and during PCR. The error rate of the proof-reading enzyme Phusion High-Fidelity DNA Polymerase used in all PCR reactions was calculated as <7.1 × 10^−6^ nucleotides/cycle/site.

#### Viral Dynamics in Paired Serum Samples Determined by Direct Sequencing, Clonal Sequencing, and NGS

Pairwise distance between strains (for NGS and clonal analysis, this was calculated between the most similar pretreatment and posttreatment strains) was significantly higher using direct sequencing than NGS (mean 0.221 versus 0.026, respectively; *P* = 0.0002). Using direct sequencing, evidence of a new variant was detected in 10/15 (66%) patients posttreatment (Supporting Fig. 1). However, when NGS-derived pretreatment sequences were compared with clonal sequence analysis of posttreatment samples, 100% of patients had evidence of a similar variant present in pre- and posttreatment samples. A new variant (in addition to a preexisting variant) was detected in posttreatment samples in 6/15 (40%) patients (Table 1; Supporting Fig. 2A-C). This was a minority variant in three patients and a majority variant in three patients.

#### Mixed Strain Infections

All patients had evidence of multiple strain infection with 2-6 variants of genotype 1a. Seven patients had evidence of mixed subtype or genotype infection at baseline; six patients had two subtypes (1a and 1b) and one patient had a mixed genotype infection (1a and 4d). Minority strains that emerged following therapy ranged from 3% to 13% of the viral population in pretreatment samples, and reached up to 75-100% of the total viral population in posttreatment samples (Table 1).

**Table 1:** Characteristics of Viral Population Dynamics and Treatment Response in Patients With Treatment Failure

ID	Clinical Outcome	Pairwise Distance (Sanger)*	Pairwise Distance (NGS)†	New Dominance‡	New Variants§	Final Conclusion
P38	Null response	0.19	0.08	-	1	Persistent infection (New variant detected)
P63	Null response	0.03	0	-	0	Persistent infection
P67	Null response	0.04	0.04	-	0	Persistent infection
P81	Null response	0.48	0.06	13%	1	Persistent infection (New dominance and new variant detected)
P112	Null response	0.17	0.01	-	0	Persistent infection
P118	Null response	0.47	0.01	3%	0	Persistent infection (New dominance)
P21	Partial response	0.27	0	NA	1	Persistent infection (New dominance and new variant detected)
P31	Partial response	0.08	0.08	-	0	Persistent infection
P105	Partial response	0.46	0	-	1	Persistent infection (New variant detected)
P75	Relapse	0	0	-	1	Persistent infection (New variant detected)
P76	Relapse	0.24	0.05	3.2%	0	Persistent infection (New dominance)
P101	Relapse	0	0	-	0	Persistent infection
P57	Relapse	0.33	0.03	9%	0	Persistent infection (New dominance)
P131	Relapse	0.27	0.05	-	2	Persistent infection (New variant detected)
P141	Relapse	0.24	0.01	3.9%	0	Persistent infection (New dominance)

* Outcome is determined by comparing consensus sequence of pre- and posttreatment samples using Sanger sequencing.

† Pairwise distance is the pairwise distance between the similar variants in paired samples where a new dominance of pre-exisiting minority strain was noticed.

‡ New dominance is the frequency of the new dominant variant of the posttreatment sample detected in the pretreatment sample.

§ Number of new variants detected in posttreatment sample.

#### Patient Groups

**Fig. 1:**
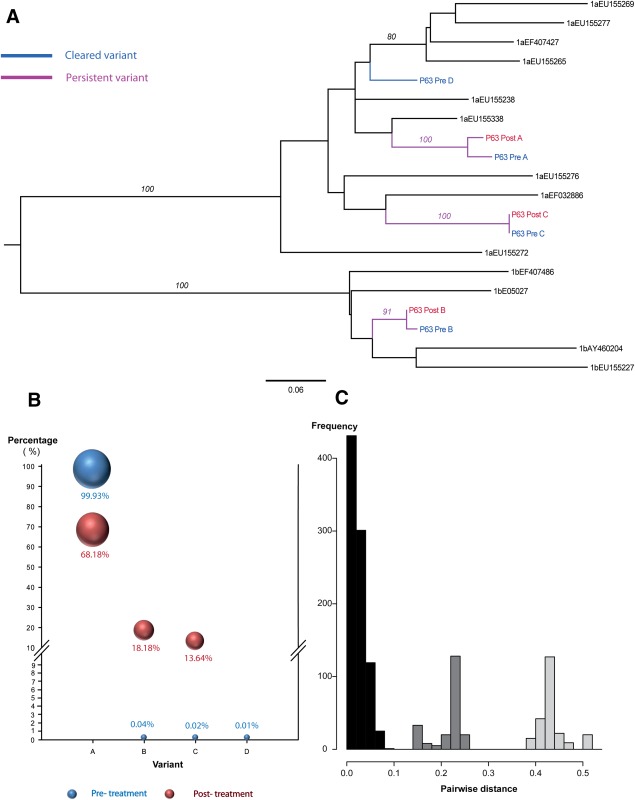
Comparison of viral complexity in paired serum samples (pre- and posttreatment) in patient -P63, Null response, Persistent infection. (A) A Maximum likelihood tree was constructed using nucleotide sequences from paired samples and selected HCV reference sequences for the Los Alamos HCV database. A total of 4 (A-D) HCV variants detected. The analysis included; 25 clonal sequences (posttreatment), and 46156 reads derived from 454 pyrosequencing (pretreatment). There were a total of 183 positions in the final dataset. (B) Bubble chart of the frequency of each variant (A-D) in pre- and posttreatment samples. (C) Pairwise distance between the most similar variants in the pre- and posttreatment samples (p-distance).

In null responders (six patients: P63, P38, P67, P81, P112 and P118; Fig. 1; Supporting Figs. 3-7), mixed subtype infection (1a/1b) at the outset was detected in 5/6 patients, and the sixth patient had multiple variants of genotype 1a. All six patients had evidence of a similar strain present pre- and postinfection. Three patients (P63, P67, P112) had persistent infection with the same pretreatment dominant strain, one patient (P118) had new dominance of a preexisting minority variant, and two patients (P81, P38) had evidence of a new variant in addition to preexisting strains (minority and majority posttreatment variant, respectively).

**Fig. 2:**
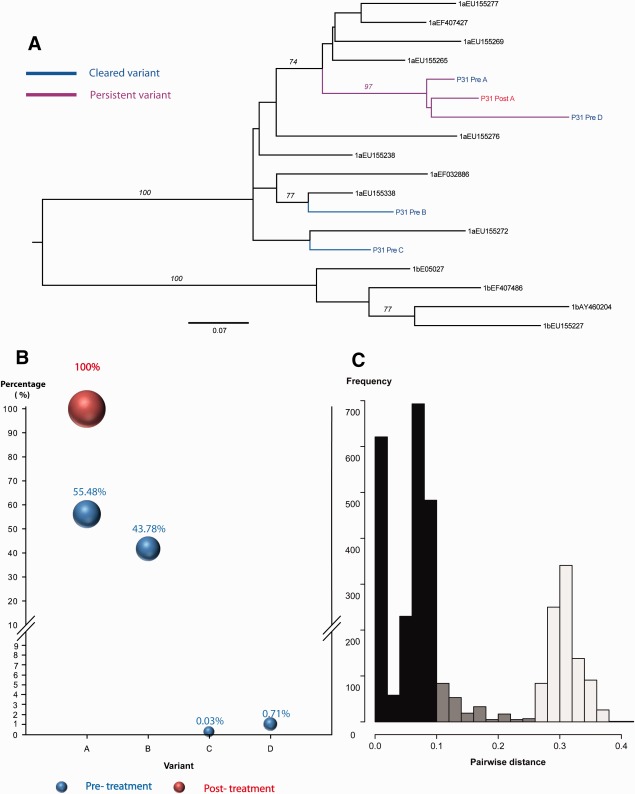
Comparison of viral complexity in paired serum samples (pre- and posttreatment) in patient -P31, Partial response, Persistent infection. (A) A Maximum likelihood tree was constructed using nucleotide sequences from paired samples and selected HCV reference sequences for the Los Alamos HCV database. A total of 4 (A-D) HCV variants detected. The analysis included; 35 clonal sequences (posttreatment), and 36422 reads derived from 454 pyrosequencing (pretreatment). There were a total of 183 positions in the final dataset. (B) Bubble chart of the frequency of each variant (A-D) in pre- and posttreatment samples. (C) Pairwise distance between the most similar variants in the pre- and posttreatment samples (p-distance).

In all partial responders (P31, P21, P105; Fig. 2; Supporting Figs. 8, 9), multiple variants were present (3, 4, and 5 variants, respectively). One patient had persistent infection with the same pretreatment variant (P31) and two patients (P21, P105) had persistent infection with evidence of a new previously unidentified strain in the posttreatment sample (minority and majority variants, respectively).

**Fig. 3:**
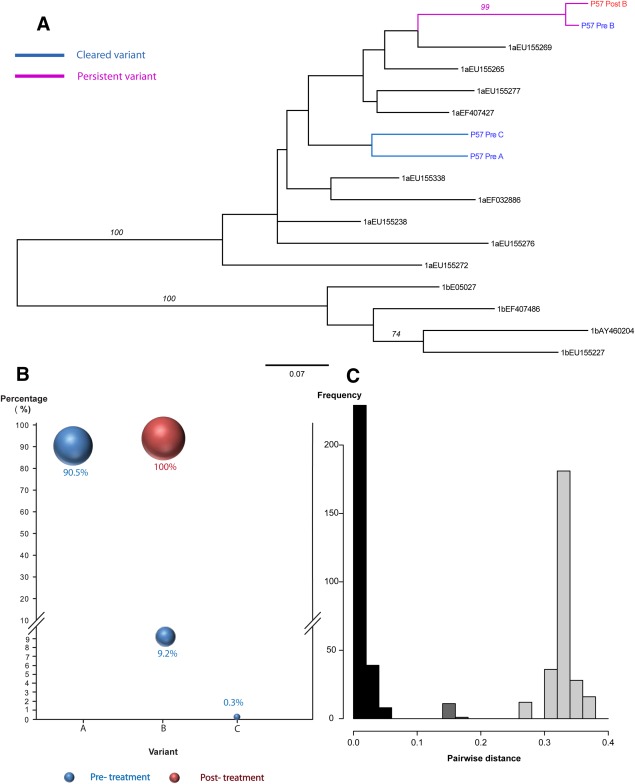
Comparison of viral complexity in paired serum samples (pre- and posttreatment) in patient -P57, Relapse, Persistent infection with new dominance of pre-existing minority variant. (A) A Maximum likelihood tree was constructed using nucleotide sequences from paired samples and selected HCV reference sequences for the Los Alamos HCV database. A total of 3 (A-C) HCV variants detected. The analysis included; 20 clonal sequences (posttreatment), and 23042 reads derived from 454 pyrosequencing (pretreatment). There were a total of 183 positions in the final dataset. (B) Bubble chart of the frequency of each variant (A-C) in pre- and posttreatment samples. (C) Pairwise distance between the most similar variants in the pre- and posttreatment samples (p-distance).

In relapsers (six patients: P57, P141, P76, P75, P101, P131; Fig. 3; Supporting Figs. 10-14), all patients had evidence of persisting variants and four of them (P101, P57, P76, P141) showed new dominance of preexisting minority strains. Two patients had evidence of new previously undetected strains; in one case the previously undetected variant became dominant in the posttreatment sample (P131) while in another patient (P75), the undetected variant was a minority strain (29%) on top of a preexisting variant (71%).

## Discussion

There have to date been few detailed studies of HCV infection and reinfection in the setting of antiviral treatment in HIV-infected MSM. Behavioral studies have shown that HIV-infected patients with acute HCV are likely to be at high risk of reexposure and therefore the presence of switching genotype or viral rebound is often assumed to be secondary to reinfection.^17^,^18^ Through detailed virological characterization using next-generation pyrosequencing in a prospectively sampled cohort, our study provides a new insight into viral dynamics during treatment failure in this cohort of patients.^19^,^20^

The reported prevalence of multiple strain infection in HIV and HCV coinfected subjects has been reported in up to 40% of those infected.^10^ Such estimates may be low, however, because screening methods used in most studies lack sensitivity for the detection of viruses present at low levels. Moreover, transient infections may be missed if sampling is infrequent. We used direct sequence analysis, clonal analysis, and NGS of samples obtained pre- and posttreatment to investigate whether treatment failure occurred most commonly due to viral recrudescence or reinfection.

In contrast to other studies, our findings indicate that multiple infections are common in early HCV infection, reaching 100% in our cohort, with a mean of 3.8 variants present prior to treatment.^21^ Other studies may not have detected multiple strains because of limited sampling or because of primer selection bias, for example, employing only genotype 1- and 2-specific primer sets; deep sequencing has higher sensitivity in detecting minor variants that are missed by conventional methods. In addition, it is also possible that our cohort harbor more HCV strains, as patients that have failed treatment may have a more diverse quasispecies.

The detection of multiple viral strains at a single pretreatment timepoint in patients with acute HCV could be the result of simultaneous transmission, or superinfection within a short timeframe. Mixed HCV infection has previously been reported as being transient, as a consequence of a more effective immune response against one viral strain, or competition between variants with the fitter strain having an advantage over others.^22^ We found that although a dominant variant was present in most samples, mixed infection was present at both timepoints examined in the majority of our patients, and variation in quasispecies composition was common over time, suggesting that certain strains may have been positively selected during treatment. Strikingly, we found preexisting strains present in 100% of patients sampled following treatment failure. This finding suggests that clearance and reinfection is not the commonest mechanism of treatment failure in this cohort.

This is in keeping with a lack of evidence of reinfection in other cohorts of HIV-infected patients with chronic HCV, although in this acutely infected cohort, evidence of genotype or subtype switching was seen more frequently.^23^ This could be attributed to the sensitivity of deep sequencing in differentiating variants of the same genotype compared to Sanger sequencing or PCR-based hybridization assays.

The presence of previously undetected variants in 40% of posttreatment samples could represent superinfection, but could also represent variants present below the threshold of detection using pyrosequence analysis. This phenomenon occurred in those with a null response, partial response, and following relapse.

In null responders, all patients had a persistent strain pre- and posttreatment and three of six patients had a persistent variant that remained dominant throughout. Emerging dominance of a preexisting minority variant occurred in two patients, one of whom had evidence of a previously undetected minority variant following treatment. In the remaining patient, a new majority variant emerged after treatment in addition to a persistent minority variant. This could represent superinfection or emergence of a strain below the limit of detection of pyrosequencing (in this case, 46,755 sequence reads were analyzed pretreatment).

In partial responders, we found evidence of persistent variants in all three patients. In two patients (P21 and P105), the dominant variant cleared following treatment, while the third patient (P31) cleared a variant representing 44% of the pretreatment viral population. In all cases, clearance was concurrent with a fall in viral load during treatment. A previously undetected variant was present in two of three patients in posttreatment samples. Such variants may have been positively selected from minority variants undetected in the pretreatment sample or could represent superinfection during treatment.

In patients with viral relapse, all patients had evidence of a preexisting variant present in posttreatment samples. Three patients (P57, P76, and P141) had evidence of emerging dominance of preexisting minority strains (rising from 3-9% of in pretreatment samples to 100% in posttreatment samples). Two patients (P101 and P75) had the same majority variant present pre- and posttreatment. In P75, a previously undetected minority variant was also detected posttreatment. One patient (P131) had evidence of a persisting minority variant and in addition, two new variants were detected. We considered this to be the most likely case of superinfection in the cohort.

**Fig. 4:**
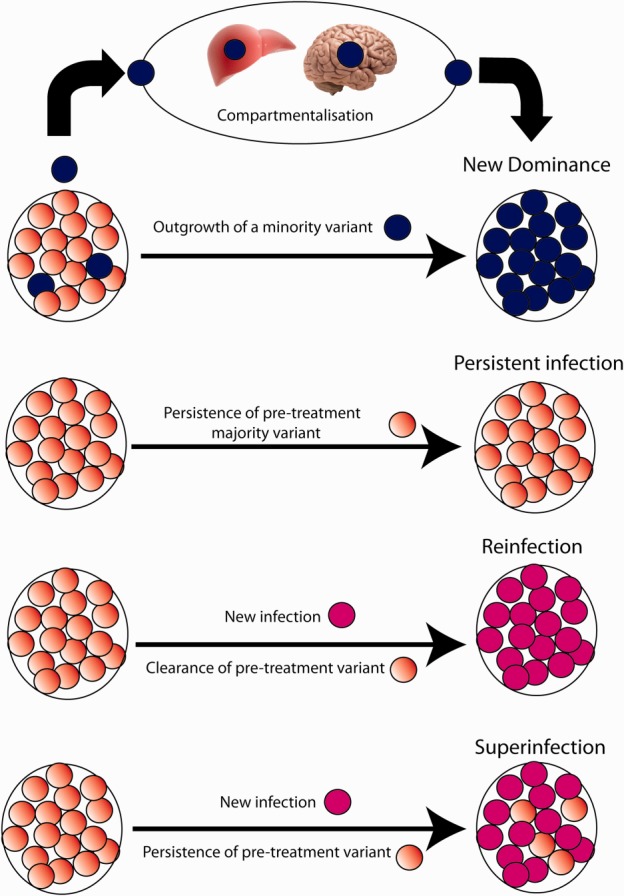
Viral dynamics during treatment failure.

In this study, NGS revealed that persisting strains were present in 100% of patients with treatment failure. If Sanger sequencing had been used alone, we would have detected persisting strains in only 34% of cases. We have shown that when a new variant is detected using direct Sanger sequencing, this usually represents the emergence of a minority variant already present in the pretreatment sample (Fig. 4). It is likely that such emergent variants represent viral strains with reduced sensitivity to antiviral medications. We found no evidence of reinfection in this cohort, despite the likelihood of ongoing behavioral risk. We cannot, however, exclude the possibility of patients being reinfected from the same source, and superinfection could have occurred in as many as 6/15 (40%) of the patient cohort in whom a previously undetected (new) variant was found. In three of these cases (20%), the new variant represented the majority variant and in three cases (20%), the new variant was a minority strain. The presence of new variants in posttreatment samples could also represent previously undetected minority variants selected by treatment or compartmentalized strains within different regions of the liver, lymphocytes, or the central nervous system.^10^,^24^–^27^ The role of compartmentalized virus acting as a reserve for future viral rebound is, as yet, relatively unexplored. It has been postulated as a strong and independent predictor of treatment efficacy.^28^ Hara et al.^29^ demonstrated that in late relapsers, HCV strains could be detected at low levels in liver biopsies during the aviremic phase, in keeping with the possibility of compartmentalization in patients with viral relapse.

Several studies of reinfection following treatment-induced clearance have previously been limited by incomplete longitudinal follow-up and insensitive detection methods.^30^ Lambers et al.^5^ described an alarmingly high incidence rate of sexually transmitted HCV reinfection of 15.2 per 100 person years among HIV-infected MSM previously successfully treated for primary HCV infection. Martin et al.^6^ also described a high risk of HCV reinfection among HIV-positive MSM who were either treated for or who spontaneously cleared initial HCV infection. In a German PWID, a reinfection rate of 0-4.1/100 person-years has been proposed.^31^ These studies did not incorporate data from studies designed to identify mixed strain infections prior to treatment, nor emergence of minority variants following treatment. It is likely that the majority of recorded reinfections were preexisting infections that became detectable after a dominant strain had cleared. A limitation of our work is that we did not investigate late relapse (viral rebound more than 24 weeks after the end of treatment) in this study; in other HCV populations, this has been shown to be a result of persistent infection^32^; this would be an interesting area of further study in this population, as it is clear that patients in this and similar cohorts are at ongoing high risk of reinfection.^18^ We propose, however, that the definition of reinfection, persisting infection, or superinfection should always be based on rigorous viral sequencing techniques.

In conclusion, deep sequencing technologies are a powerful tool for obtaining a more accurate insight into the dynamics of variants in the HCV quasispecies in human samples. The detection of multiple genotypes that have the potential to emerge following treatment may also have implications in the new era of DAAs when the presence of multiple genotypes and low-level resistance mutations may impact on treatment success.

## Abbreviations

DAA: direct-acting antiviral
HCV: hepatitis C virus
HIV: human immunodeficiency virus
IFN: interferon
MSM: men who have sex with men
RBV: ribavirin
SVR: sustained virological response
