# Supplementary material for: Next-Generation Sequencing Sheds Light on the Natural History of Hepatitis C Infection in Patients Who Fail Treatment
Source: Hepatology. 2014 Jul 30;61(1):88–97. doi: 10.1002/hep.27192 (PMC4303934; doi:10.1002/hep.27192)
Supplement: Supplementary file 1 — Supporting Information [file hep0061-0088-sd1.pdf]

Supplementary Table 1. Clinical characteristics of patient cohort

ID	Age	CD4	Nadir CD4	Route of transmission	IL28B	RVR	HAART	SVR
**P21**	36	770	60	UAI	-	-	Yes	No
**P31**	35	510	580	UAI	-	-	No	No
**P38**	31	830	320	UAI, INDU, PWID	CT	No	Yes	No
**P57**	46	710	20	UAI, INDU, PWID	CC	No	Yes	No
**P63**	47	400	150	UAI, INDU, PWID	CT	No	Yes	No
**P67**	40	340	80	UAI, INDU, PWID	CC	No	Yes	No
**P75**	32	220	220	UAI, INDU	TT	No	Yes	No
**P76**	48	390	210	UAI	-	Yes	Yes	No
**P81**	53	510	260	UAI	-	-	Yes	No
**P101**	35	640	380	UAI, INDU	TT	No	Yes	No
**P105**	39	790	300	UAI	CC	No	Yes	No
**P112**	46	220	130	UAI	-	No	No	No
**P118**	28	500	290	UAI, INDU	CT	No	Yes	No
**P131**	50	680	340	UAI	CT	Yes	Yes	No
**P141**	21	560	420	UAI	-	No	No	No

UAI – Unprotected Anal Intercourse, PWID – People Who Inject Drugs, INDU – Intranasal Drug Use, RVR – Rapid Virological Response.

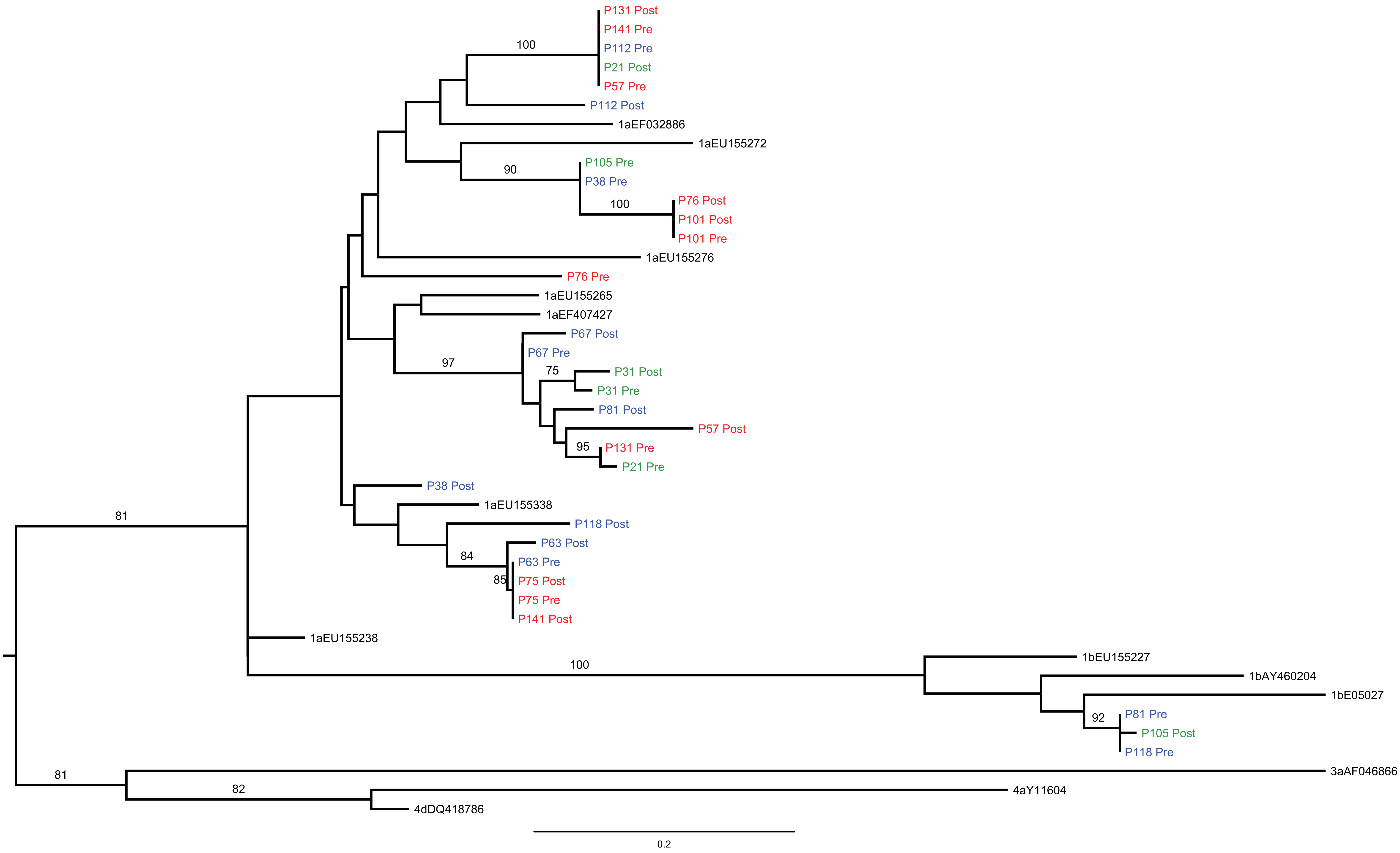

Supplementary figure 1. Maximum likelihood phylogenetic tree of direct Sanger sequences pre and post-treatment A maximum likelihood tree was constructed using nucleotide sequences determined using Sanger sequencing from paired samples and selected HCV reference sequences downloaded from the Los Alamos HCV database

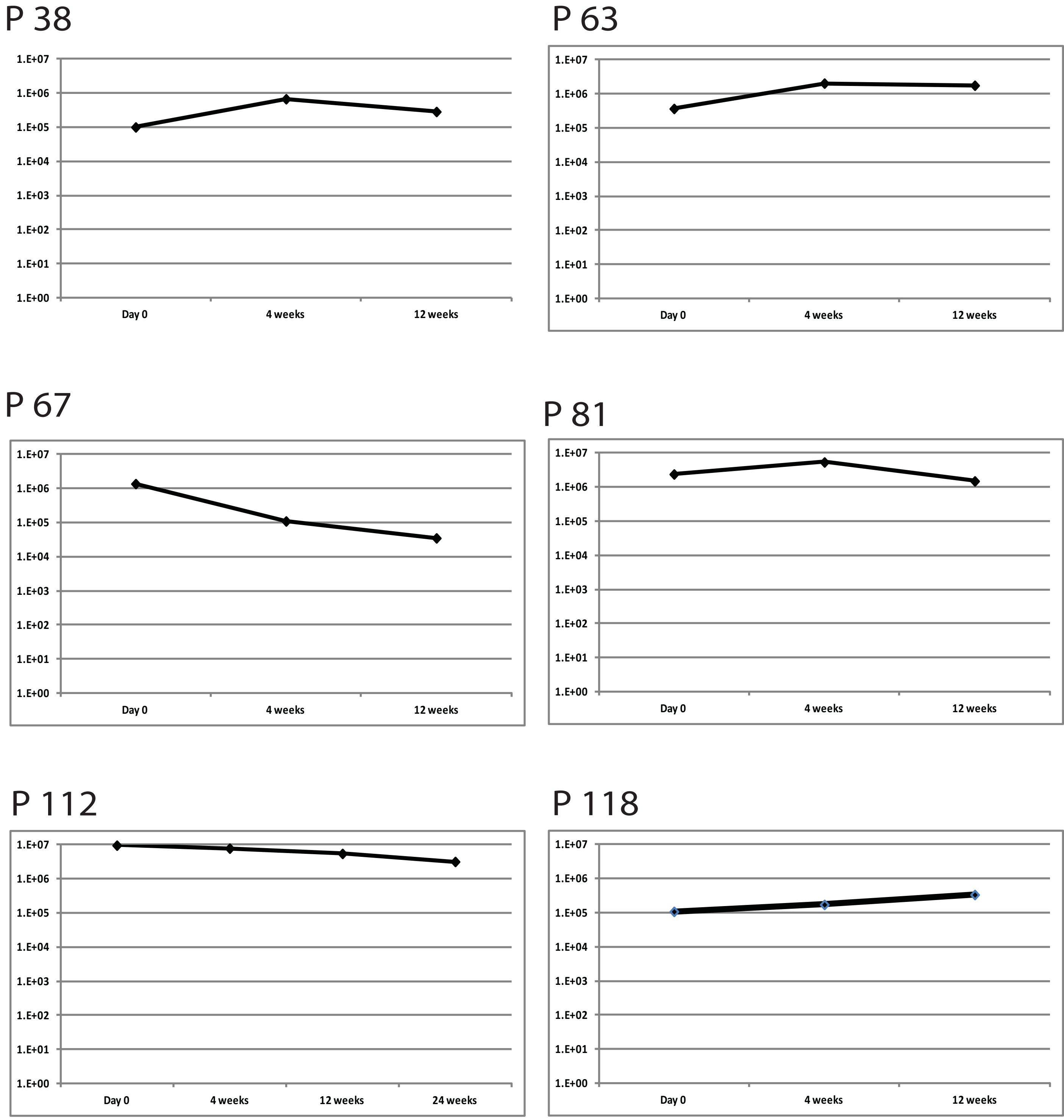

Supplementary figure 2-A The viral load dynamics since start of treatment (Day 0), during the treatment and post treatment in null responders. Viral load values in IU/ml.

P 21

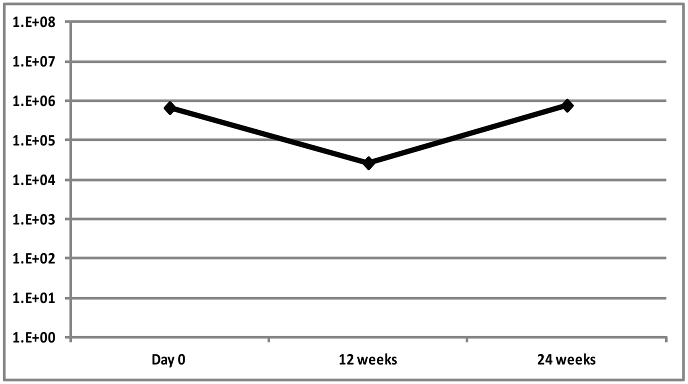

P 31

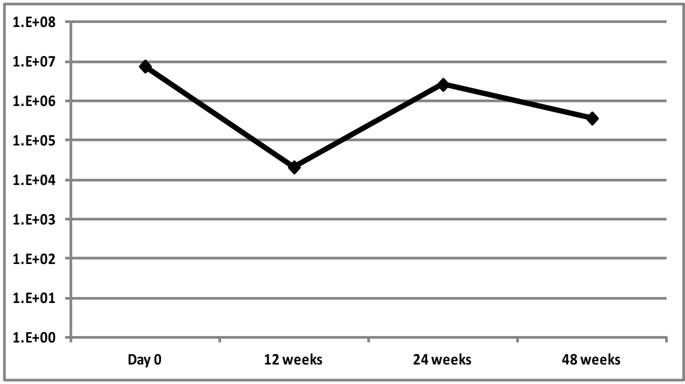

P 105

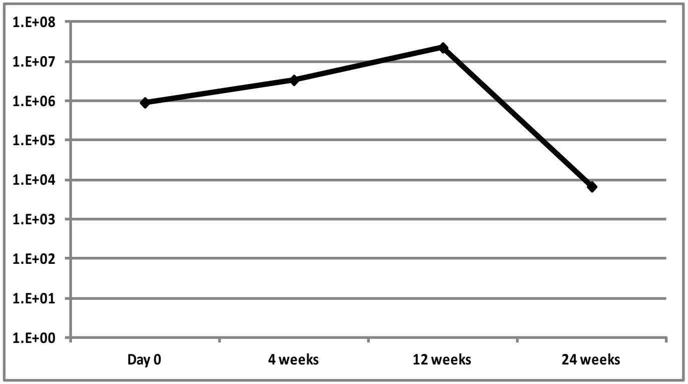

Supplementary figure 2-B The viral load dynamics since start of treatment (Day 0), during the treatment and post treatment in partial responders. (4 weeks) time point was not measured in all patients. Viral load values in IU/ml.

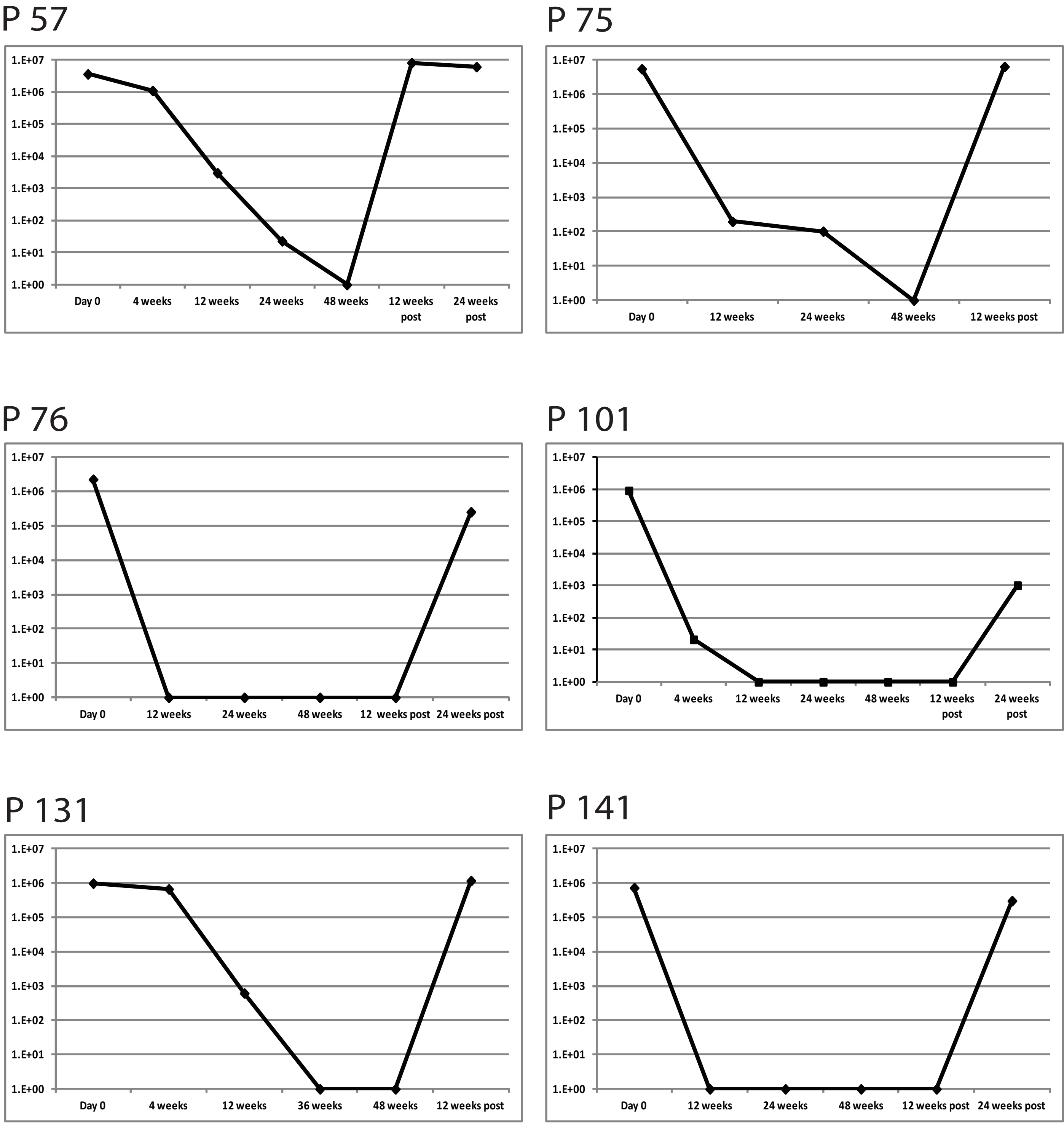

Supplementary figure 2-C The viral load dynamics since start of treatment (Day 0), during the treatment and post treatment in relapsers. (4 weeks) time point was not measured in all patients. Viral load values in IU/ml.

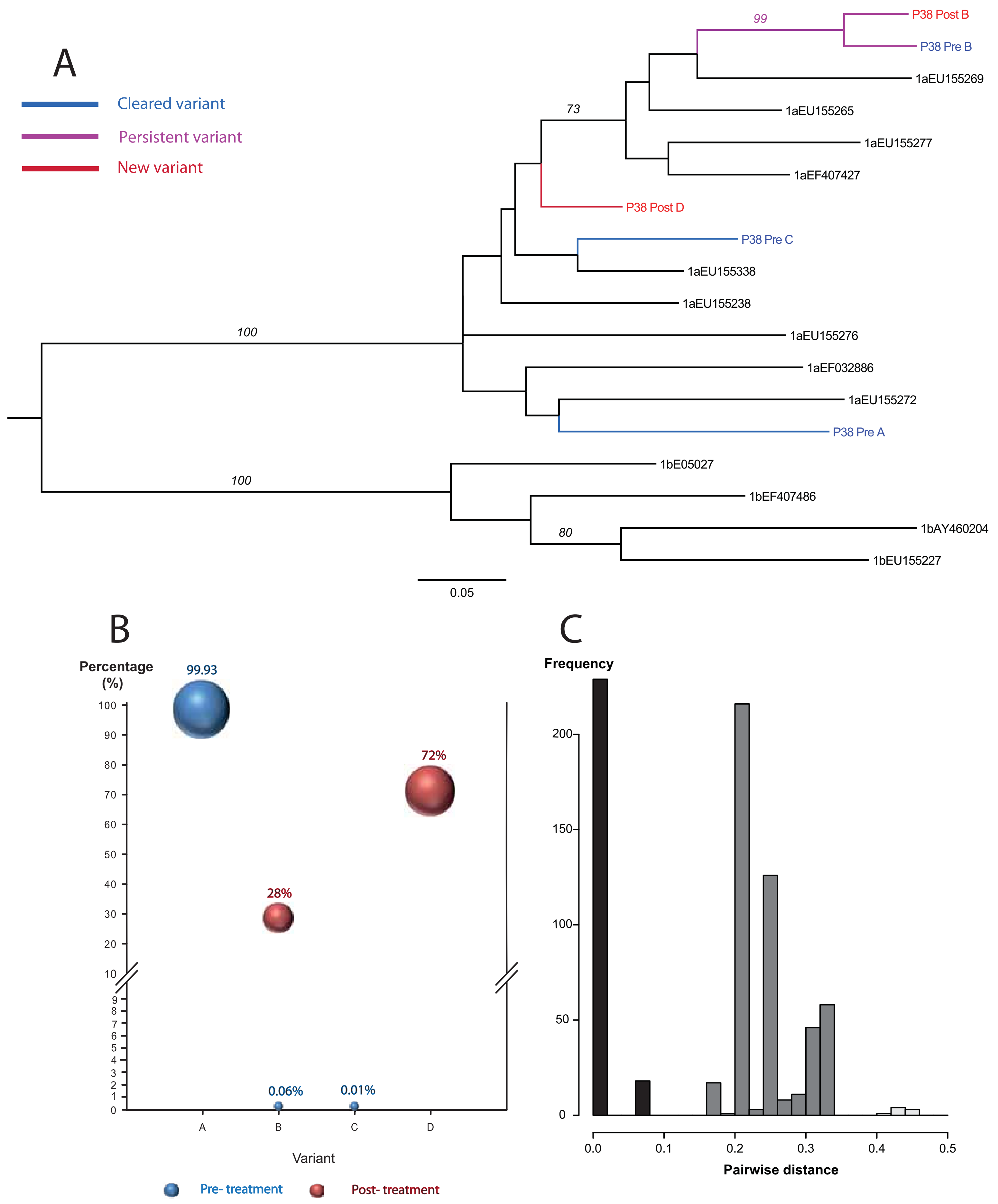

Supplementary figure 3. Comparison of viral complexity in paired serum samples (pre- and post-treatment) in patient -P38, (Null response, Persistent infection with new post-treatment variant detected.
(**A**) A Maximum likelihood tree was constructed using nucleotide sequences from paired samples and selected HCV reference sequences for the Los Alamos HCV database. A total of 4(A-D) HCV variants detected. The analysis included; 25 clonal sequences (post-treatment), and 46755 reads derived from 454 pyrosequencing (pre-treatment). There were a total of 183 positions in the final dataset. (**B**) Bubble chart of the frequency of each variant (A-D) in pre- and post-treatment samples. (**C**) Pairwise distance between the most similar variants in the pre- and post-reatment samples (p-distance).

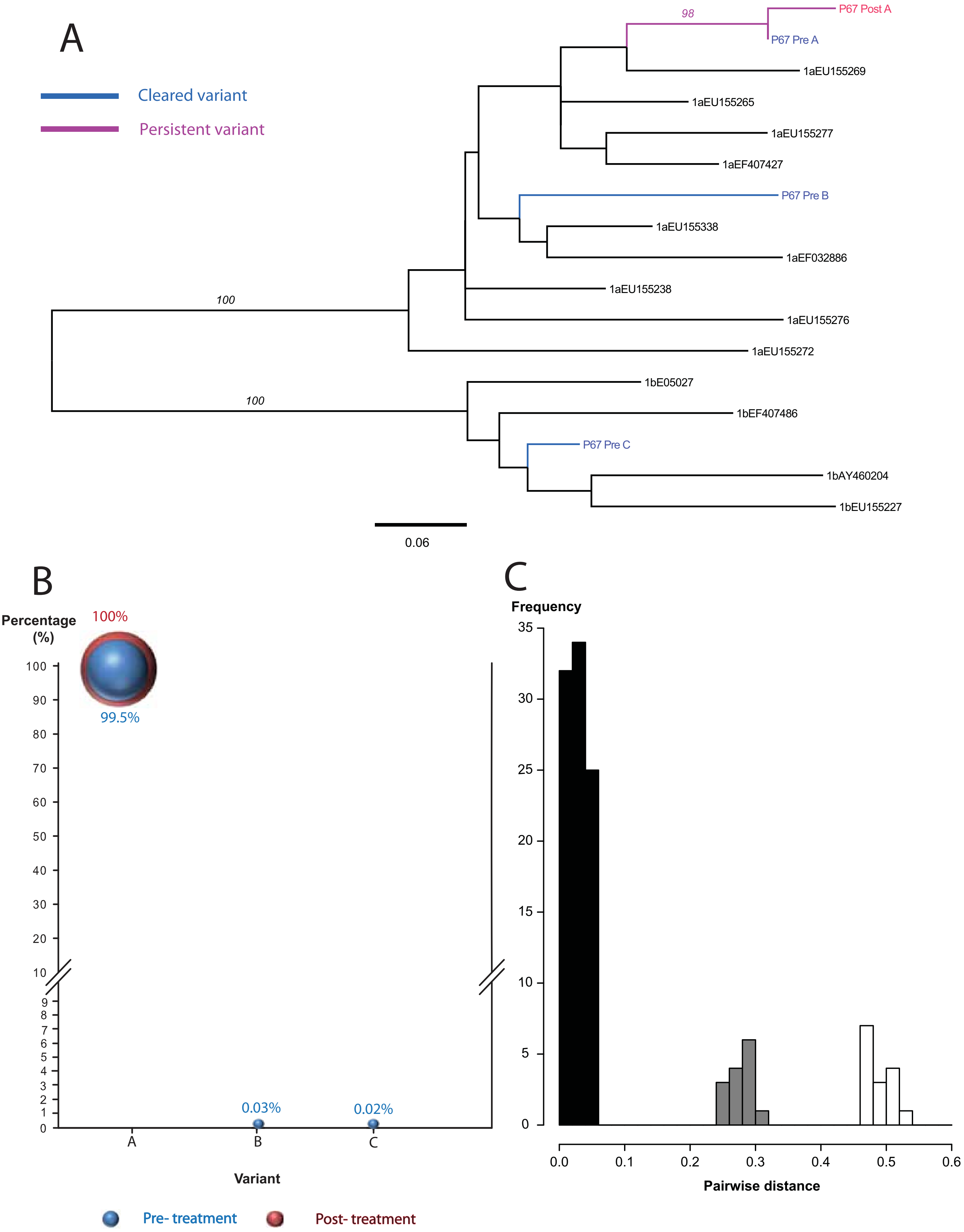

Supplementary figure 4. Comparison of viral complexity in paired serum samples (pre- and post-treatment) in patient -P67, (Null response, Persistent infection).
(**A**) A maximum likelihood tree was constructed using nucleotide sequences from paired samples and selected HCV reference sequences for the Los Alamos HCV database. A total of 3(A-C) HCV variants detected. The analysis included; 8 clonal sequences (post-treatment), and 10742 reads derived from 454 pyrosequencing (pre-treatment). There were a total of 183 positions in the final dataset. (**B**) Bubble chart of the frequency of each variant (A-C) in pre- and post-treatment samples. (**C**) Pairwise distance between the most similar variants in the pre- and post-reatment samples (p-distance).

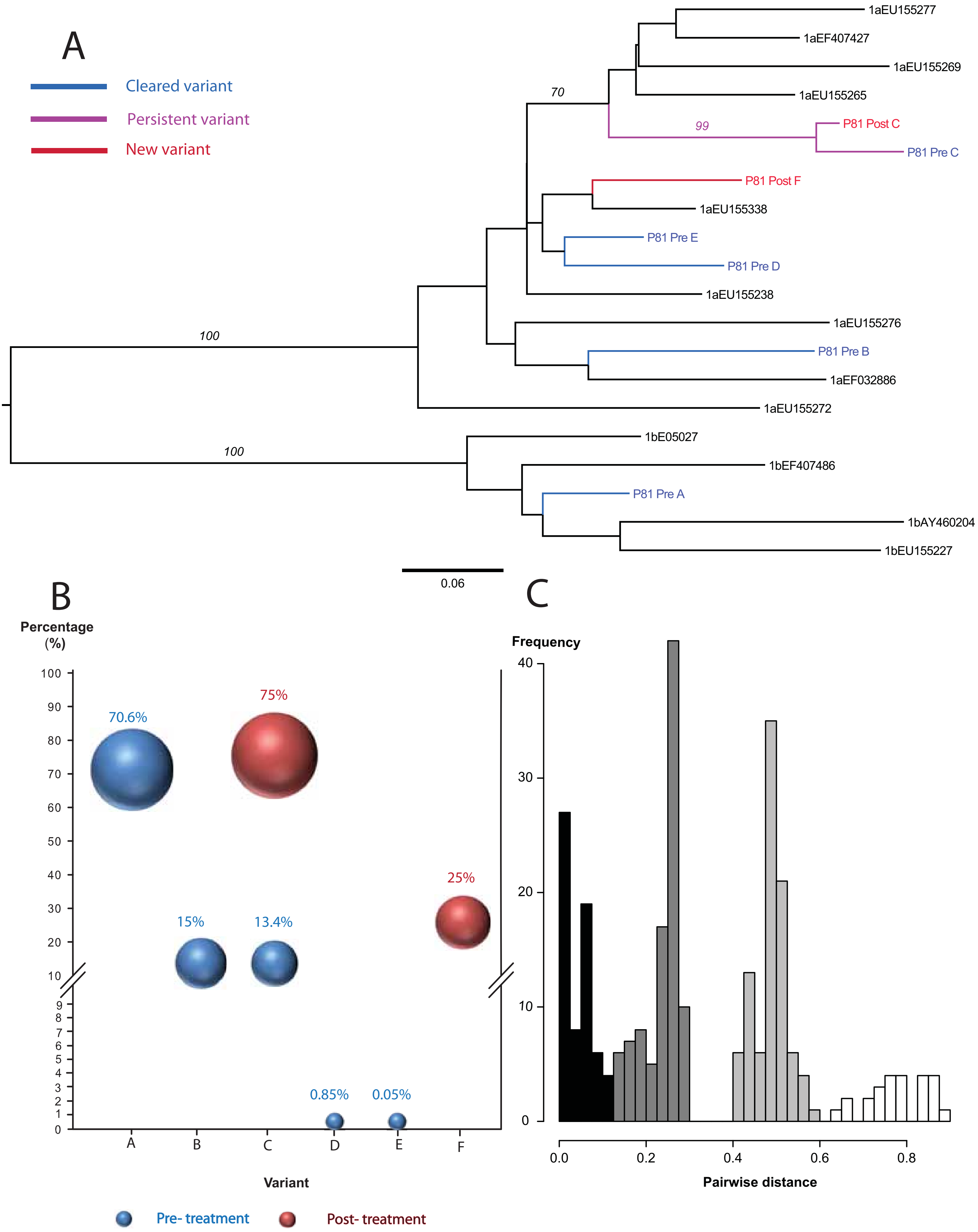

Supplementary figure 5. Comparison of viral complexity in paired serum samples (pre- and post-treatment) in patient -P81, (Null response, Persistent infection with a new post-treatment variant detected).
(**A**) A maximum likelihood tree was constructed using nucleotide sequences from paired samples and selected HCV reference sequences for the Los Alamos HCV database. A total of 6(A-F) HCV variants detected. The analysis included; 12 clonal sequences (post-treatment), and 19610 reads derived from 454 pyrosequencing (pre-treatment). There were a total of 183 positions in the final dataset. (**B**) Bubble chart of the frequency of each variant (A-F) in pre- and post-treatment samples. (**C**) Pairwise distance between the most similar variants in the pre- and post-reatment samples (p-distance).

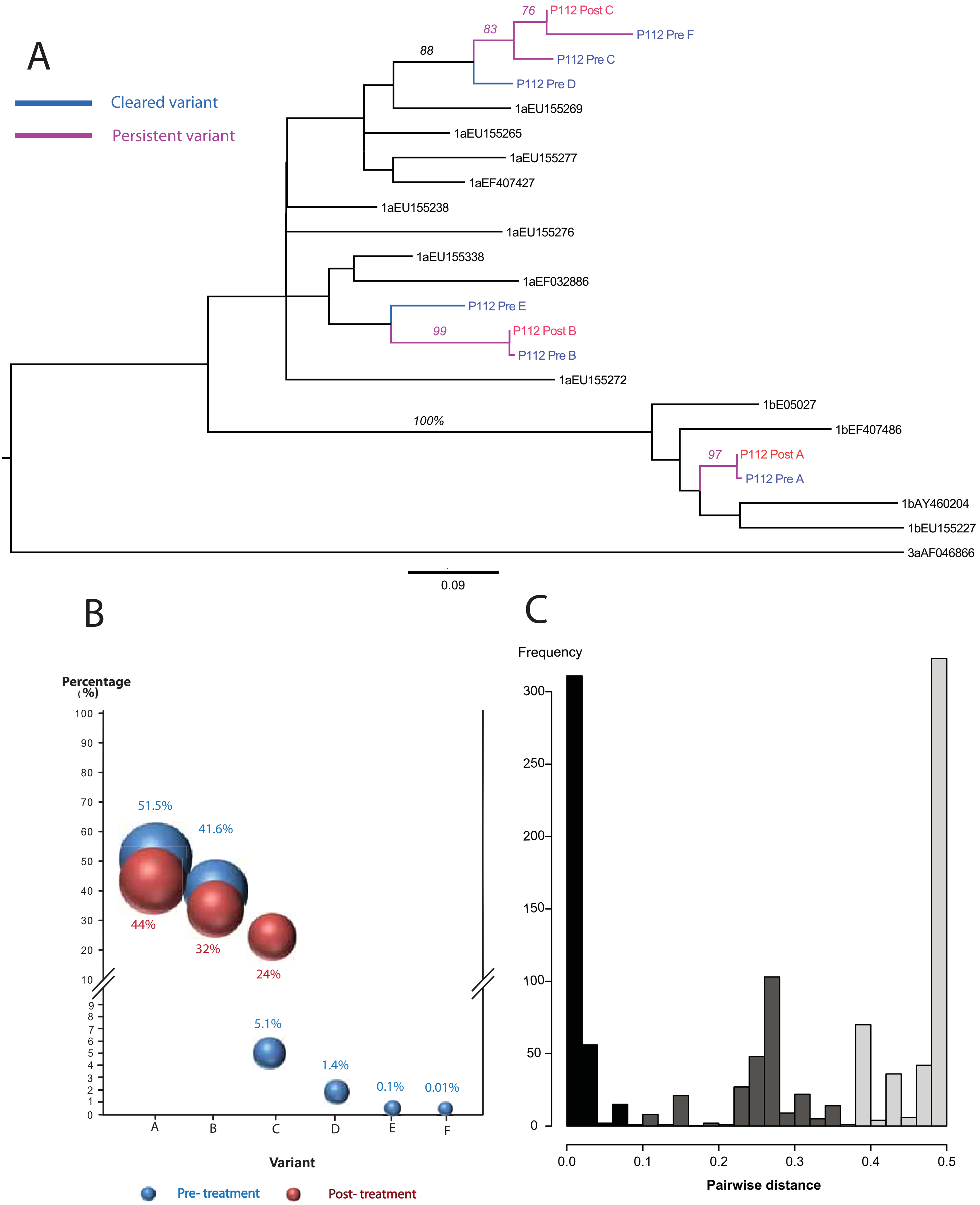

Supplementary figure 6. Comparison of viral complexity in paired serum samples (pre- and post-treatment) in patient -P112, (Null response, Persistent infection).
(**A**) A maximum likelihood tree was constructed using nucleotide sequences from paired samples and selected HCV reference sequences for the Los Alamos HCV database. A total of 6(A-F) HCV variants detected. The analysis included; 25 clonal sequences (post-treatment), and 21246 reads derived from 454 pyrosequencing (pre-treatment). There were a total of 183 positions in the final dataset. (**B**) Bubble chart of the frequency of each variant (A-F) in pre- and post-treatment samples. (**C**) Pairwise distance between the most similar variants in the pre- and post-reatment samples (p-distance).

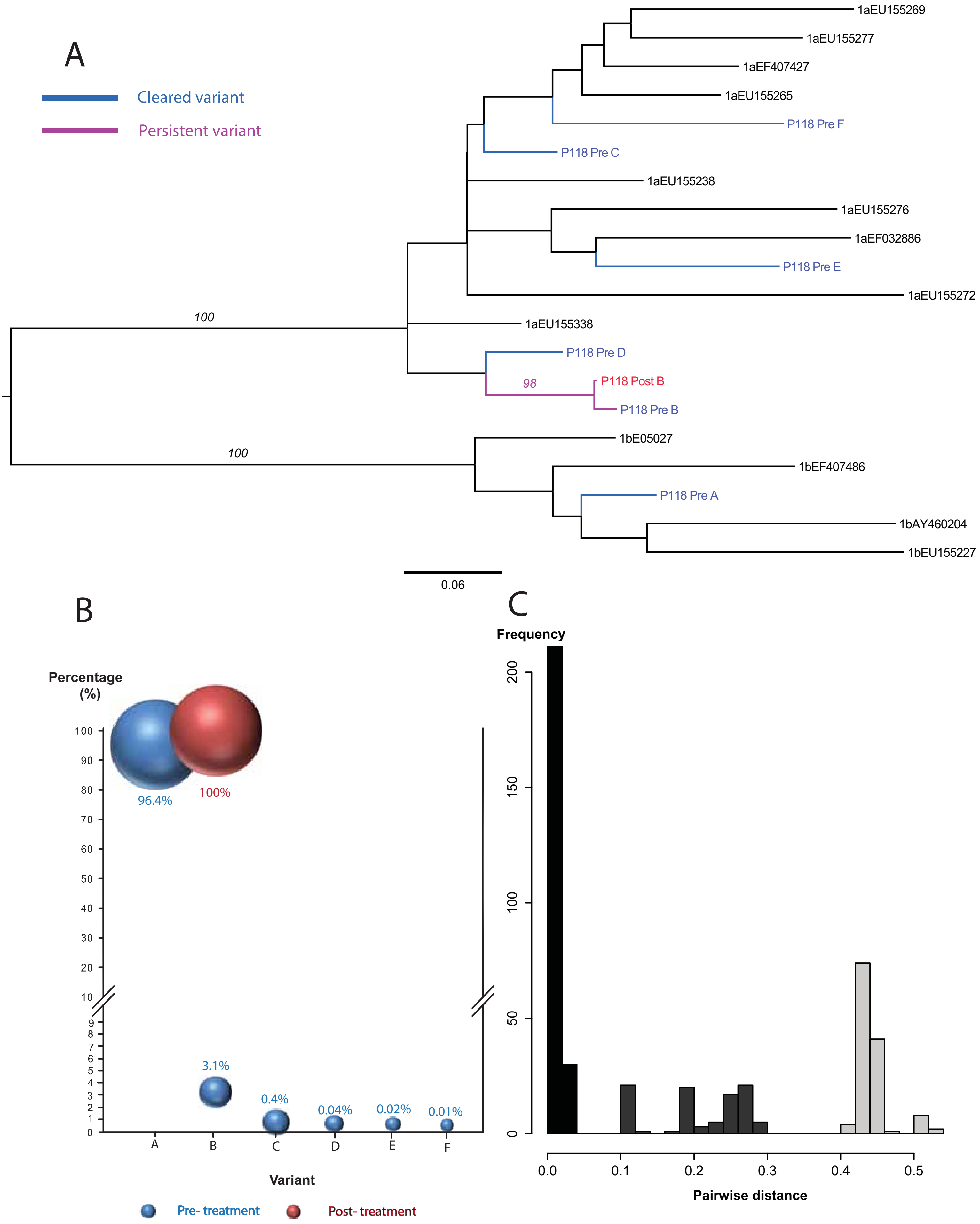

Supplementary figure 7. Comparison of viral complexity in paired serum samples (pre- and post-treatment) in patient -P118, (Null response, Persistent infection with new dominance of a pre-existing minority variant).
(**A**) A maximum likelihood tree was constructed using nucleotide sequences from paired samples and selected HCV reference sequences for the Los Alamos HCV database. A total of 6(A-F) HCV variants detected. The analysis included; 20 clonal sequences (post-treatment), and 28131 reads derived from 454 pyrosequencing (pre-treatment). There were a total of 183 positions in the final dataset. (**B**) Bubble chart of the frequency of each variant (A-F) in pre- and post-treatment samples. (**C**) Pairwise distance between the most similar variants in the pre- and post-reatment samples (p-distance).

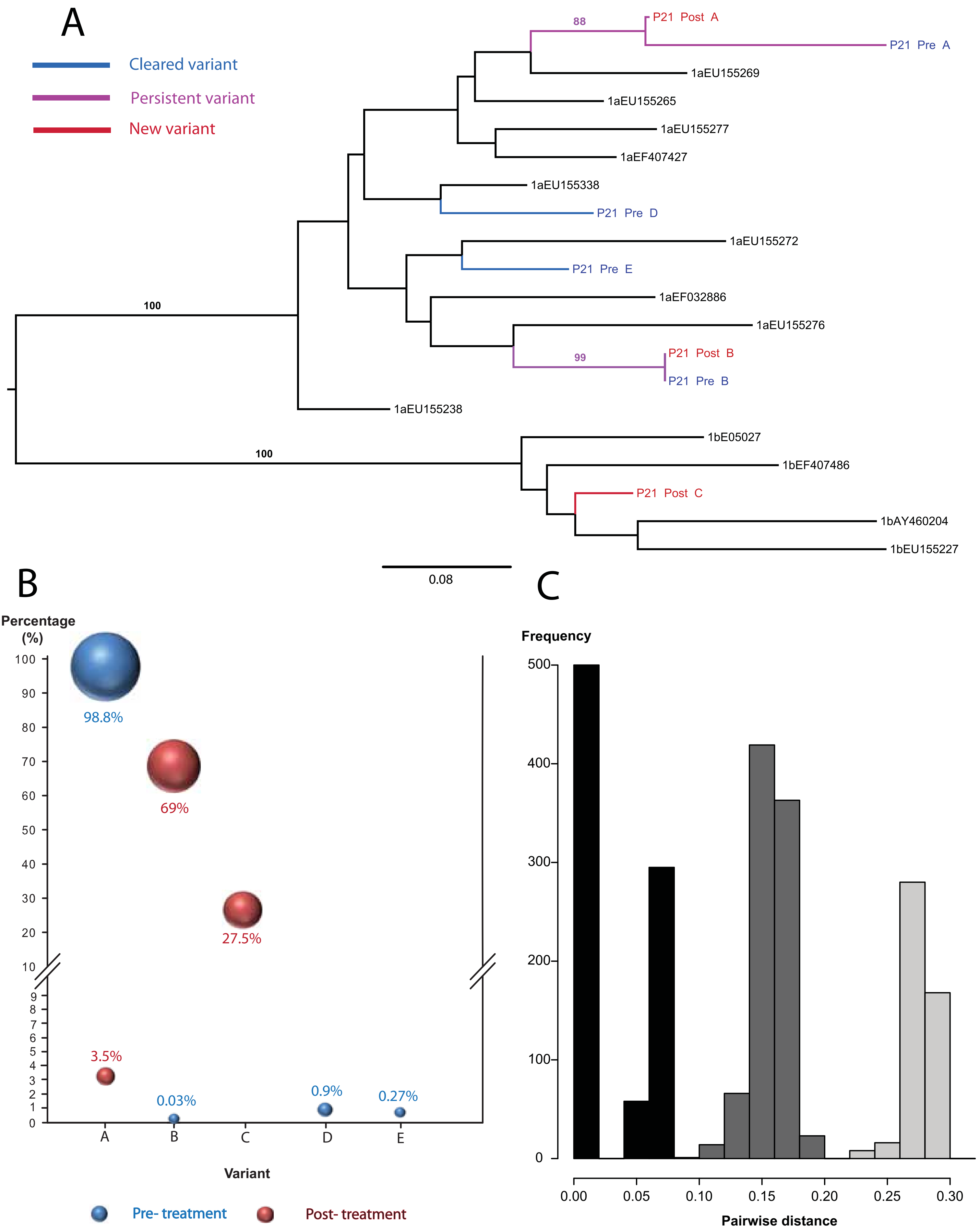

Supplementary figure 8. Comparison of viral complexity in paired serum samples (pre- and post-treatment) in patient -P21, (Partial response, Persistent infection with new post-treatment variant detected).

(**A**) A maximum likelihood tree was constructed using nucleotide sequences from paired samples and selected HCV reference sequences for the Los Alamos HCV database. A total of 5(A-E) HCV variants detected. The analysis included; 29 clonal sequences (post-treatment), and 46755 reads derived from 454 pyrosequencing (pre-treatment). There were a total of 183 positions in the final dataset. (**B**) Bubble chart of the frequency of each variant (A-E) in pre- and post-treatment samples. (**C**) Pairwise distance between the most similar variants in the pre- and post-reatment samples (p-distance).

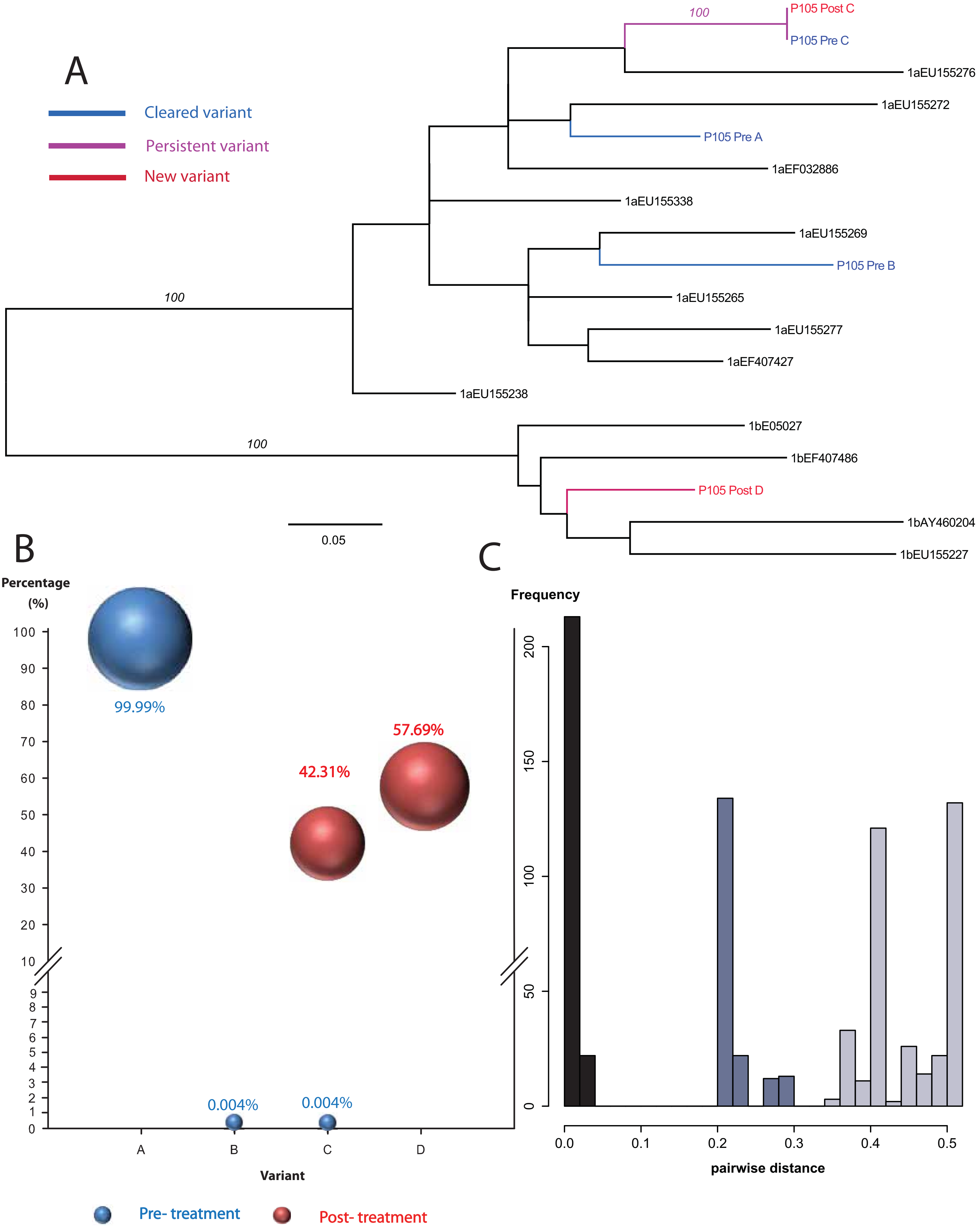

Supplementary figure 9. Comparison of viral complexity in paired serum samples (pre- and post-treatment) in patient -P105, (Partial response, Persistent infection with expansion of a pre-existing minority variant with a new post-treatment variant detected).

(**A**) A maximum likelihood tree was constructed using nucleotide sequences from paired samples and selected HCV reference sequences for the Los Alamos HCV database. A total of 4(A-D) HCV variants detected. The analysis included; 26 clonal sequences (post-treatment), and 44296 reads derived from 454 pyrosequencing (pre-treatment). There were a total of 183 positions in the final dataset. (**B**) Bubble chart of the frequency of each variant (A-D) in pre- and post-treatment samples. (**C**) Pairwise distance between the most similar variants in the pre- and post-reatment samples (p-distance).

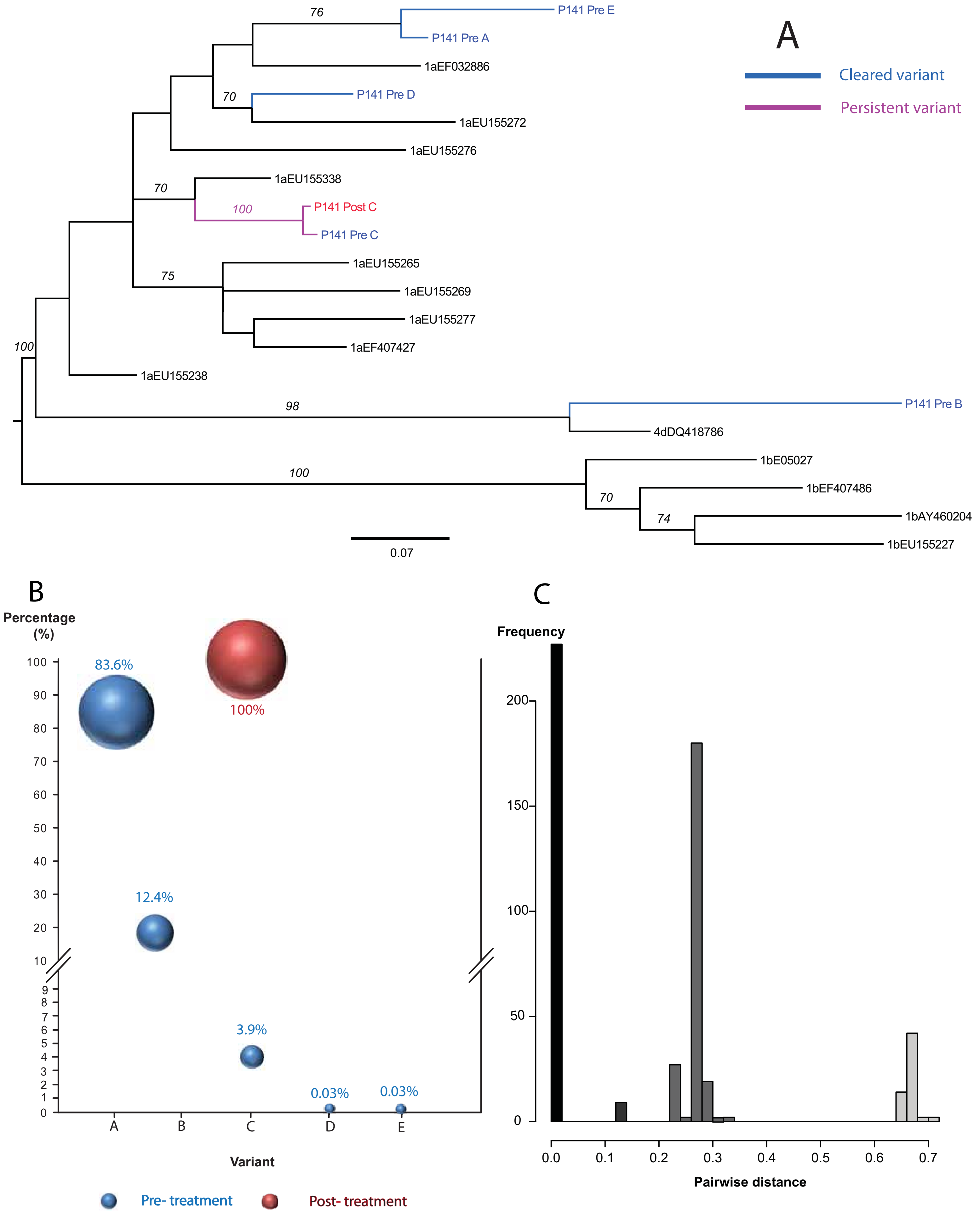

Supplementary figure 10. Comparison of viral complexity in paired serum samples (pre- and post-treatment) in patient -P141, (Relapse, Persistent infection with new dominance of pre-existing minority variant).
(**A**) A maximum likelihood tree was constructed using nucleotide sequences from paired samples and selected HCV reference sequences for the Los Alamos HCV database. A total of 5(A-E) HCV variants detected. The analysis included; 18 clonal sequences (post-treatment), and 23588 reads derived from 454 pyrosequencing (pre-treatment). There were a total of 183 positions in the final dataset. (**B**) Bubble chart of the frequency of each variant (A-E) in pre- and post-treatment samples. (**C**) Pairwise distance between the most similar variants in the pre- and post-reatment samples (p-distance).

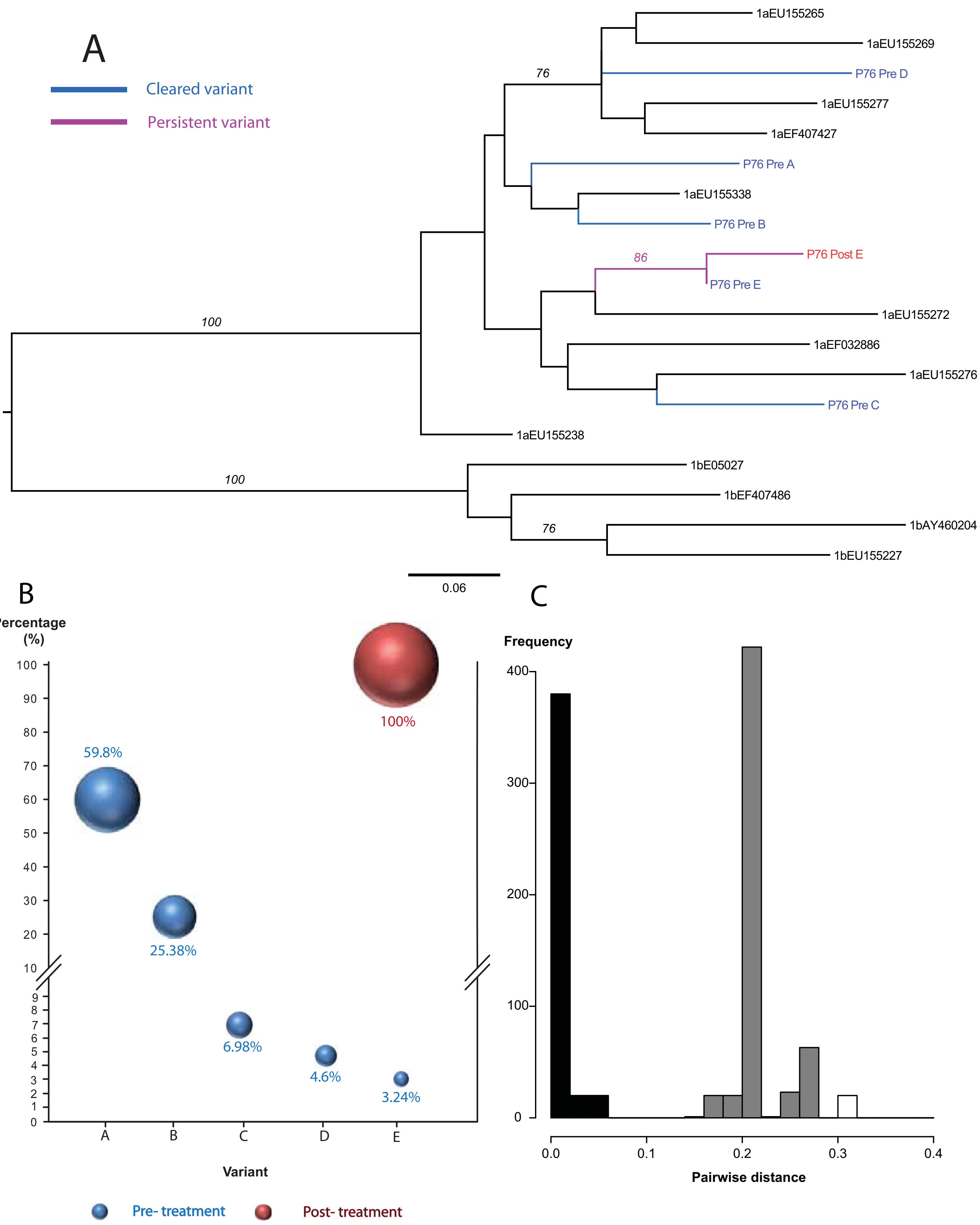

Supplementary figure 11. Comparison of viral complexity in paired serum samples (pre- and post-treatment) in patient -P76, (Relapse, Persistent infection with new dominance of pre-existing minority variant).
(**A**) A Maximum likelihood tree was constructed using nucleotide sequences from paired samples and selected HCV reference sequences for the Los Alamos HCV database. A total of 4(A-E) HCV variants detected. The analysis included; 20 clonal sequences (post-treatment), and 587 reads derived from 454 pyrosequencing (pre-treatment). There were a total of 183 positions in the final dataset. (**B**) Bubble chart of the frequency of each variant (A-E) in pre- and post-treatment samples. (**C**) Pairwise distance between the most similar variants in the pre- and post-reatment samples (p-distance).

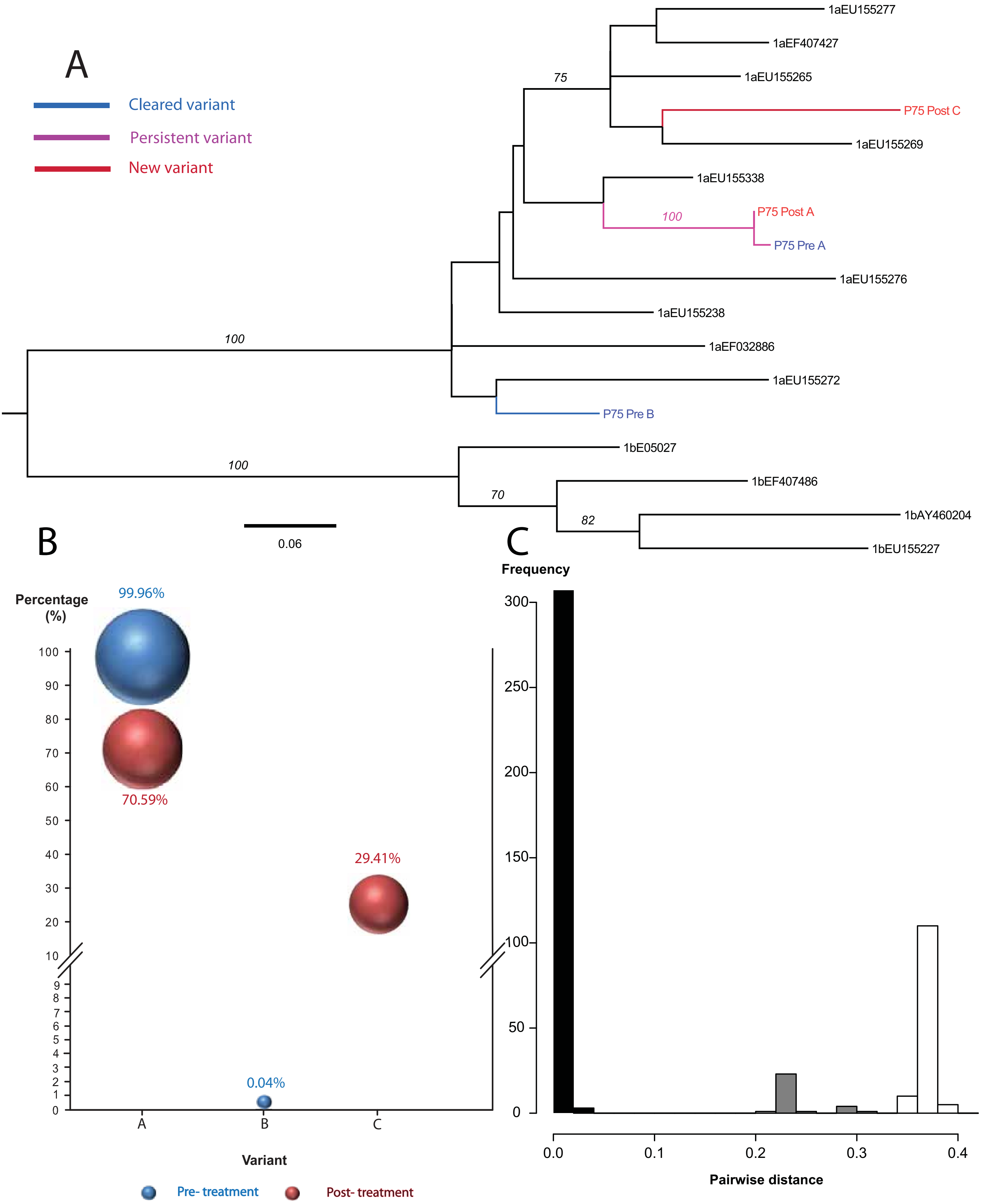

Supplementary figure 12. Comparison of viral complexity in paired serum samples (pre- and post-treatment) in patient -P75, (Relapse, Persistent infection with new post-treatment variant detected).
(**A**) A Maximum likelihood tree was constructed using nucleotide sequences from paired samples and selected HCV reference sequences for the Los Alamos HCV database. A total of 3(A-C) HCV variants detected. The analysis included; 17 clonal sequences (post-treatment), and 23639 reads derived from 454 pyrosequencing (pre-treatment). There were a total of 183 positions in the final dataset. (**B**) Bubble chart of the frequency of each variant (A-C) in pre- and post-treatment samples. (**C**) Pairwise distance between the most similar variants in the pre- and post-reatment samples (p-distance).

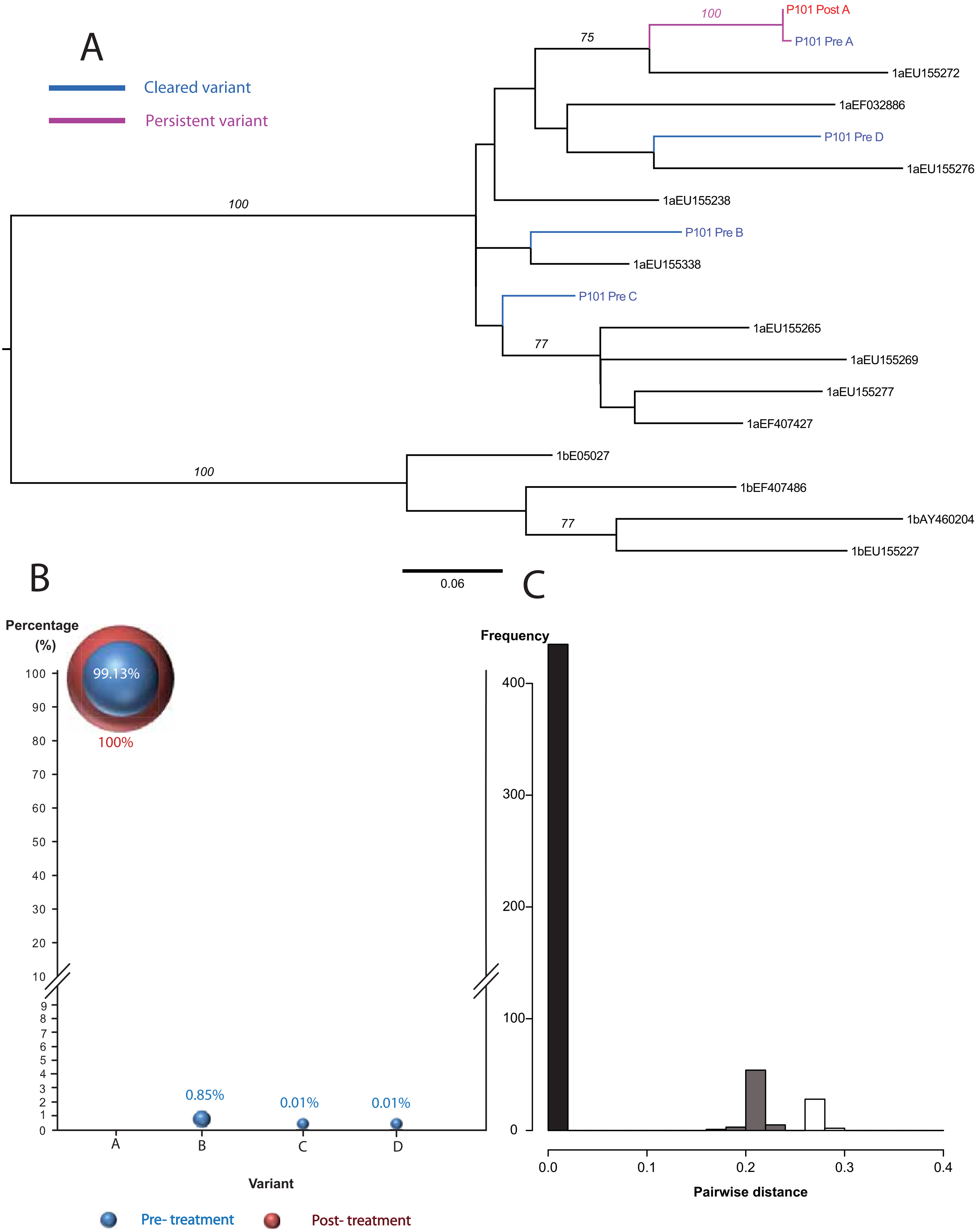

Supplementary figure 13. Comparison of viral complexity in paired serum samples (pre- and post-treatment) in patient -P101, (Relapse, Persistent infection).
(**A**) A maximum likelihood tree was constructed using nucleotide sequences from paired samples and selected HCV reference sequences for the Los Alamos HCV database. A total of 4(A-D) HCV variants detected. The analysis included; 24 clonal sequences (post-treatment), and 21265 reads derived from 454 pyrosequencing (pre-treatment). There were a total of 183 positions in the final dataset. (**B**) Bubble chart of the frequency of each variant (A-D) in pre- and post-treatment samples. (**C**) Pairwise distance between the most similar variants in the pre- and post-reatment samples (p-distance).

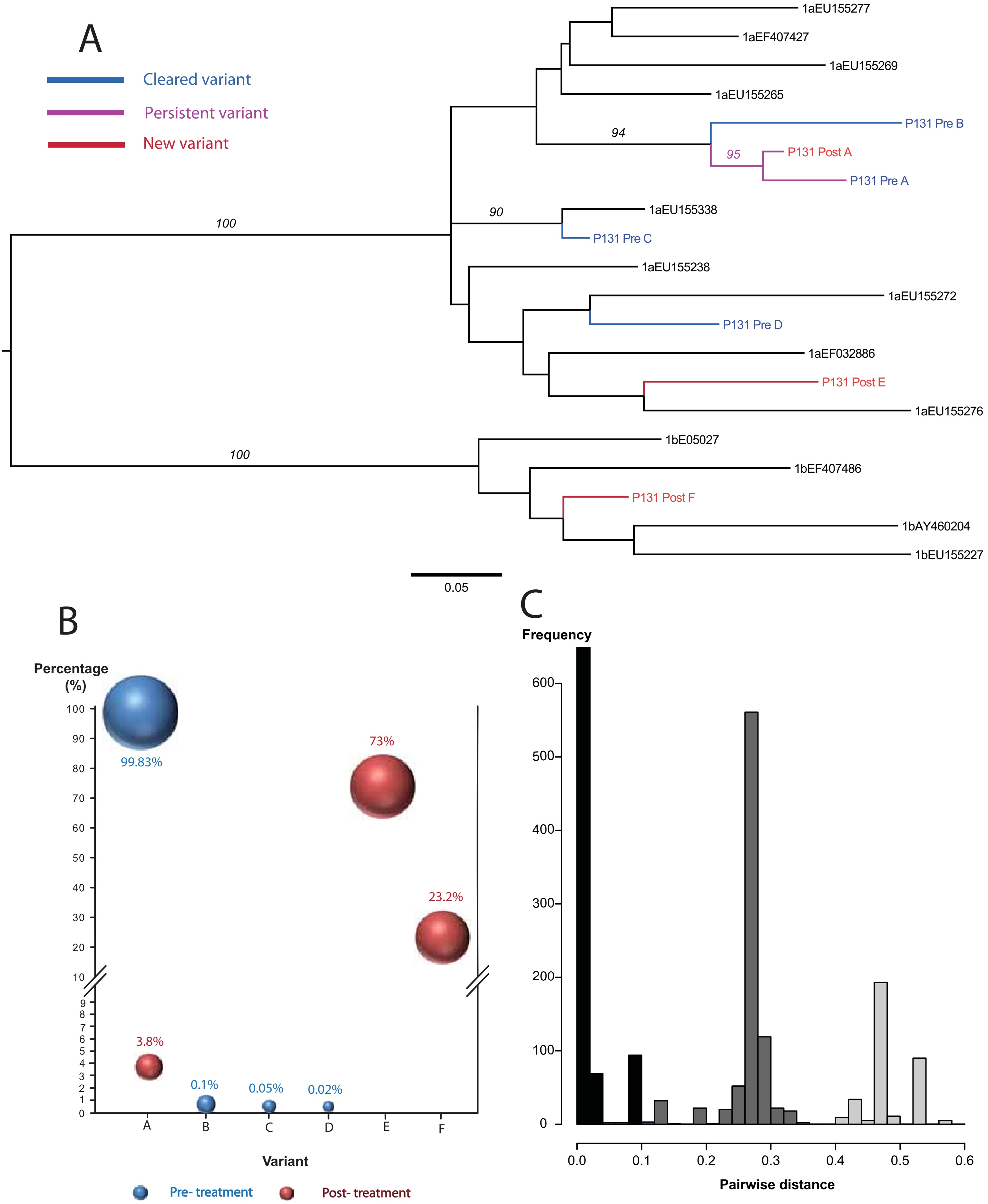

Supplementary figure 14. Comparison of viral complexity in paired serum samples (pre- and post-treatment) in patient -P131, (Relapse, Persistent infection with new post-treatment variant detected.

(**A**) A maximum likelihood tree was constructed using nucleotide sequences from paired samples and selected HCV reference sequences for the Los Alamos HCV database. A total of 6 (A-F) HCV variants detected. The analysis included; 26 clonal sequences (post-treatment), and 19739 reads derived from 454 pyrosequencing (pre-treatment). There were a total of 183 positions in the final dataset. (**B**) Bubble chart of the frequency of each variant (A-F) in pre- and post-treatment samples. (**C**) Pairwise distance between the most similar variants in the pre- and post-reatment samples (p-distance).
